# An overview and history of the Global Neurodegeneration Proteomics Consortium (GNPC)

**DOI:** 10.1002/alz70856_098477

**Published:** 2025-12-24

**Authors:** Farhad Imam

**Affiliations:** ^1^ Gates Ventures, Seattle, WA, USA

## Abstract

**Background:**

Limited understanding of biological mechanisms behind the onset and progression of major Neurodegenerative Disorders diseases [Alzheimer's Disease (AD), Parkinsons's Disease (PD), Amyotrophic Lateral Sclerosis (ALS), and Frontotemporal Dementia (FTD)] has been a burden for the discovery of novel biomarkers and treatments. Large, harmonized, patient‐derived datasets will be key in unraveling the complex biology leading to neurodegeneration. The Global Neurodegeneration Proteomics Consortium (GNPC), a public‐private partnership, undertook the legal and technical work to address these challenges and created one of the largest proteomic data sets in the world with **∼300 million unique protein measures** from, at inception, **23 partners** and nearly **40,000 biosample analyses** with an associated **50 clinical data features**.

**Method:**

The GNPC first worked to harmonize 23 individual datasets with heterogeneous clinical and proteomic data. Developing the GNPC's large data set required addressing several barriers to open data and data sharing including legal, technological, and incentive and norms‐based barriers. The complete harmonized dataset was securely provided to consortium members via the Alzheimer's Disease Data Initiative's online platform, the AD Workbench for data refinement and analysis. After one year of embargoed intra‐consortium analysis, the HDS will be made accessible by the broader research community as a **shared, global resource (July 2025)**.

**Result:**

The first version of the Harmonized Dataset (HDS) includes approximately 40,000 unique proteomic analyses on the SomaScan 5k and/or 7k proteomics array platform, in addition to a smaller subset studied with Olink and mass spectrometry methods. All samples are accompanied by 50 phenotypic indicators including demographic, cognitive data, clinical scores, and disease‐specific genotype. Central hosting of de‐identified and harmonized data occurs within the secure AD Workbench environment, including scalable compute resources for >100 active researchers.

**Conclusion:**

With over 300,000,000 unique protein measures, the GNPC represents the one of the **largest protein biomarker discovery efforts for neurodegenerative diseases**—or any specific disease area—to date. Results of preliminary analyses from the consortium's distinct workstreams (cross‐sectional profiling, longitudinal profiling, proteogenomic mapping, multivariate prediction & modeling) will be summarized, including diagnostic and predictive proteomic biomarker signatures for pan‐neurodegeneration and disease‐specific states.

